# Climate Regimes Override Micro-Site Effects on the Summer Temperature Signal of Scots Pine at Its Northern Distribution Limits

**DOI:** 10.3389/fpls.2018.01597

**Published:** 2018-11-08

**Authors:** Jelena Lange, Allan Buras, Roberto Cruz-García, Marina Gurskaya, Risto Jalkanen, Vladimir Kukarskih, Jeong-Wook Seo, Martin Wilmking

**Affiliations:** ^1^Institute of Botany and Landscape Ecology, University of Greifswald, Greifswald, Germany; ^2^Forest Ecology and Forest Management, Wageningen University and Research, Wageningen, Netherlands; ^3^Institute of Plant and Animal Ecology, UB of the Russian Academy of Sciences, Yekaterinburg, Russia; ^4^Natural Resources Institute Finland (Luke), Rovaniemi Research Unit, Rovaniemi, Finland; ^5^Department of Wood and Paper Science, College of Agriculture, Life and Environmental Sciences, Chungbuk National University, Cheongju, South Korea

**Keywords:** *Pinus sylvestris*, tree-ring width, maximum latewood density, micro-site, climate change, climate regime, treeline, boreal forest

## Abstract

Tree growth at northern boreal treelines is generally limited by summer temperature, hence tree rings serve as natural archives of past climatic conditions. However, there is increasing evidence that a changing summer climate as well as certain micro-site conditions can lead to a weakening or loss of the summer temperature signal in trees growing in treeline environments. This phenomenon poses a challenge to all applications relying on stable temperature-growth relationships such as temperature reconstructions and dynamic vegetation models. We tested the effect of differing ecological and climatological conditions on the summer temperature signal of Scots pine at its northern distribution limits by analyzing twelve sites distributed along a 2200 km gradient from Finland to Western Siberia (Russia). Two frequently used proxies in dendroclimatology, ring width and maximum latewood density, were correlated with summer temperature for the period 1901–2013 separately for (i) dry vs. wet micro-sites and (ii) years with dry/warm vs. wet/cold climate regimes prevailing during the growing season. Differing climate regimes significantly affected the temperature signal of Scots pine at about half of our sites: While correlations were stronger in wet/cold than in dry/warm years at most sites located in Russia, differing climate regimes had only little effect at Finnish sites. Both tree-ring proxies were affected in a similar way. Interestingly, micro-site differences significantly affected absolute tree growth, but had only minor effects on the climatic signal at our sites. We conclude that, despite the treeline-proximal location, growth-limiting conditions seem to be exceeded in dry/warm years at most Russian sites, leading to a weakening or loss of the summer temperature signal in Scots pine here. With projected temperature increase, unstable summer temperature signals in Scots pine tree rings might become more frequent, possibly affecting dendroclimatological applications and related fields.

## Introduction

Growth of trees at northern boreal treelines is primarily limited by summer temperature due to the short and cool growing season prevailing in the subarctic (Körner, 1998, 2012; Holtmeier, 2009). Consequently, annually resolved tree-ring width (TRW) and maximum latewood density (MXD) from treeline conifers are regularly used as natural archives to reconstruct past summer temperatures over several centuries and sometimes millennia (e.g., Briffa et al., 1990, 2002a,b; Grudd et al., 2002; Hantemirov and Shiyatov, 2002; Linderholm and Gunnarson, 2005; Grudd, 2008; McCarroll et al., 2013; Porter et al., 2013; Matskovsky and Helama, 2014). However, large-scale studies of tree growth-climate sensitivity at treeline ecotone sites point to a non-uniform reaction of treelines to summer temperature (e.g., Harsch et al., 2009; Ohse et al., 2012; Hellmann et al., 2016), up to a regionally occurring complete loss of the summer temperature signal – a phenomenon which has been intensely discussed as the divergence problem, often attributed to changing climatic conditions (e.g., Wilmking et al., 2004; Driscoll et al., 2005; Wilmking et al., 2005; Lloyd and Bunn, 2007; D’Arrigo et al., 2008; Porter and Pisaric, 2011; Seo et al., 2011; Juday et al., 2015).

Scots pine (*Pinus sylvestris* L.) is widely distributed across the Eurasian boreal forest and survives across a broad range of ecological and climate conditions (e.g., it tolerates very dry sites, and acidic and wet peatland conditions; Ellenberg and Leuschner, 2010), making it a popular species for temperature reconstructions (e.g., Esper et al., 2002, 2016; Grudd et al., 2002; Linderholm and Gunnarson, 2005; Grudd, 2008; Helama et al., 2009; McCarroll et al., 2013; Matskovsky and Helama, 2014). Despite this ability to adapt, it was shown repeatedly that ecological micro-site conditions can affect the climatic signal of Scots pine at northern boreal treelines in different ways. Previous investigations indicate that the summer temperature signal of Scots pine in northern Fennoscandia was reduced on wet micro-sites, i.e., at lakeshores, riparian sites or peatlands, when compared to neighboring dry sites, with this response being attributed to stressful anaerobic and nutrient poor soil conditions (Linderholm, 2001; Linderholm et al., 2002, 2014; Matskovsky and Helama, 2014; Düthorn et al., 2015, 2016). Conversely in northern Sweden, Düthorn et al. (2013) have found a weaker summer temperature signal in Scots pine growing on dry sites relative to wet sites and attributed this finding to drought conditions.

In addition to these ecological effects, a climatically induced loss of summer temperature sensitivity in the second half of the 20th century has been reported for Scots pine growing in Finnish Lapland, presumably provoked by drought stress in the early growing season due to high temperatures and below-average precipitation sums (Seo et al., 2011; Franke et al., 2017). One study points to a complex interaction of both ecological and climatic effects: Only the combination of wet lakeshore conditions and cold/wet climatic conditions prevailing during the Little Ice Age led to reduced growth and a presumably weak climatic signal in Scots pine from altitudinal treeline in central Sweden, while all other combinations (wet soil – medieval warming period; dry soil – any of the two periods) showed no effect (Linderholm et al., 2014).

However, similar studies investigating both ecological and climatological effects simultaneously on temperature sensitivity of Scots pine from northern treelines are entirely missing, as are studies documenting micro-site effects from northern treelines outside Fennoscandia. Further, despite the stronger summer temperature signal of MXD over TRW (e.g., Grudd, 2008; Tuovinen et al., 2009), only one study has investigated micro-site effects on MXD of Scots pine so far (Düthorn et al., 2016). A heterogeneous response to climate due to micro-site effects might generally question the a priori suitability of Scots pine trees for temperature reconstructions and other dendroclimatological applications (e.g., Linderholm et al., 2014; Düthorn et al., 2015; Edvardsson et al., 2015; Hellmann et al., 2016).

Here, we investigated the simultaneous effect of ecological (micro-sites) and climatological differences on the summer temperature signal of Scots pine TRW and MXD on a broad spatial scale. Over the period of 1901–2013 and along a 2200 km gradient from oceanic Finland to continental Western Siberia, we analyzed a well replicated dataset of twelve Scots pine micro-sites, including dry and wet sites at the northern distribution limit of the species. We applied a novel approach to explore the effect of differing climate regimes on temperature-growth relationships by comparing dry/warm and wet/cold years that were equally distributed across the study period. In particular, we tested if there is a difference in strength and direction of temperature–growth relationships between (i) treeline and forest sites, (ii) dry and wet micro-sites, and (iii) dry/warm and wet/cold climate regimes, and their combinations. Assuming that temperature limits growth at our sites, we expect a strong and significant positive summer temperature signal at all sites, being slightly lower at forest than at treeline sites. Due to the growth-limiting role of temperature, we further hypothesize that effects of the climate regime are stronger than micro-site effects, and that the former are overall stronger at Russian sites than in Finland due to higher summer temperatures as a consequence of its continentality.

## Materials and Methods

### Site Selection and Sampling

**FIGURE 1:**
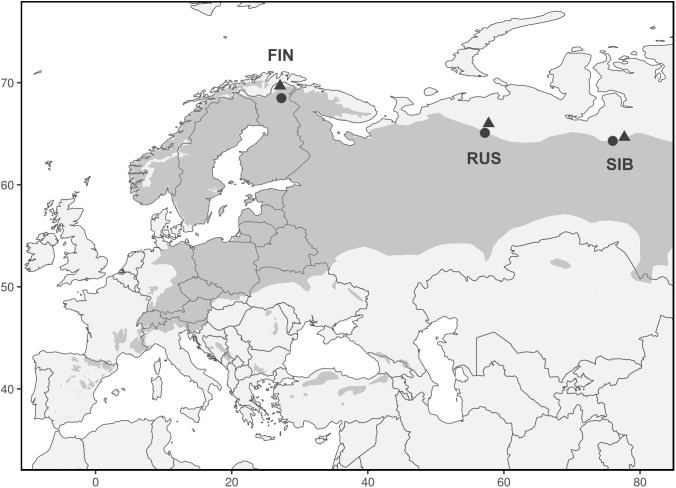
Range of *Pinus sylvestris* (dark gray) and locations of treeline (triangles) and forest (circles) sites. Each triangle and circle represents one pair of dry and wet micro-sites.

We analyzed 348 Scots pine trees from three regions at their northern distribution limit in Eurasia: Finnish Lapland (FIN), north of European Russia (RUS), and Western Siberia (SIB) (Figure 1). In each region, a treeline (“T,” located at the northern treeline or northern distribution limit of Scots pine) and a forest site (“F,” located 50–140 km south of the treeline site) were selected for sampling. At each treeline and forest site, trees from neighboring dry (“D”) and wet (“W”) micro-sites were sampled, resulting in twelve different sampling locations (Table 1). Dry micro-sites were characterized by well drained sandy soils with sparse ground cover (mainly *Cladonia* sp.), while micro-sites with peaty soil, visibly upcoming groundwater and a high abundance of indicator species for peatland (e.g., *Vaccinium* sp., *Andromeda polifolia* L.) were defined as wet sites. All Russian sites as well as the Finnish wet sites were mostly flat, ranging between 40–65 and 200–260 m a.s.l., respectively, while Finnish dry sites were located on slightly inclined south or west facing slopes (Table 1). All sites were free of permafrost. Only dominant, mature and straight growing trees without visible signs of damage or illness were selected for sampling, the typical tree form and status used for dendroclimatology. Two increment cores (A and B; 5.15 and 12 mm in diameter, respectively) were taken per tree at breast height perpendicular to each other to lower a possible bias due to irregular/eccentric growth. Diameter at breast height (dbh) and tree height were recorded for each tree.

**Table 1:** Description of the study sites and number of analyzed trees.

Region (code)	Site	Latitude	Longitude	Elevation (m.a.s.l.)	*N*	Mean age
Finland (FIN)	TD	69°41′ N	27°06′ E	200	26	190
	TW	69°41′ N	27°06′ E	200	20	190
	FD	68°28′ N	27°18′ E	270	20	301
	FW	68°30′ N	27°18′ E	260	16	261
North of European Russia (RUS)	TD	66°00′ N	57°51′ E	45	35	147
	TW	66°00′ N	57°40′ E	60	19	182
	FD	65°06′ N	57°14′ E	50	39	164^∗∗∗^
	FW	65°05′ N	57°10′ E	60	31	132^∗∗∗^
Western Siberia (SIB)	TD	64°40′ N	77°41′ E	45	29	123^∗∗^
	TW	64°40′ N	77°41′ E	40	27	183^∗∗^
	FD	64°18′ N	75°59′ E	65	29	211^∗∗∗^
	FW	64°18′ N	75°59′ E	65	28	140^∗∗∗^

*Mean tree age was tested for significant difference between each pair of dry and wet micro-sites using a Wilcoxon rank-sum test or a Student’s *t*-test (depending on whether data was normally distributed). Significant differences are marked with an asterisk (^∗^*p* < 0.05, ^∗∗^*p* < 0.01, ^∗∗∗^*p* < 0.001). Site abbreviations: T, treeline; F, forest; D, dry; W, wet*.

### Dendrochronological Methods

The surface of core A was smoothed using a WSL sledge microtome (Gärtner and Nievergelt, 2010) in order to make annual rings visible, and scanned visually using a conventional scanner. TRW was measured on the scanned image with 0.001 mm precision using the software CooRecorder v. 7.7 (Cybis Elektronik and Data AB). After securing core B to a wooden holder, laths of 1.25 ± 0.1 mm thickness were cut out of the core using a twin-bladed circular saw (Dendrocut 2003, Walesch Electronics^1^) and subsequently boiled for 24 h in a Soxhlet extractor filled with 96% Ethanol to remove resins and other soluble substances from the wood (Schweingruber, 1978). After a minimum drying and acclimatization time of 12 h under controlled air temperature (20°C) and relative humidity (50%) conditions, the laths were X-rayed under the same controlled conditions using an ITRAX MultiScanner (Cox Analytical Systems) with an exposure time of 25 ms, an intensity of 30 kV/50 mA, and in steps of 20 μm. A standard calibration plexi ladder was used to calibrate gray-level light intensity to wood density in g/cm^3^ for each X-ray scan. MXD and TRW were measured on the obtained gray-scale images using the WinDENDRO software v. 2014b (Regents Instruments Inc.). The mean thickness of each wooden lath was measured using electronic calipers and subsequently used for calibration of the respective sample to account for possible deviations in thickness among the individual laths. All TRW and MXD series were visually cross-dated in CDendro (Cybis Elektronik and Data AB). Series from blurred images and with visible growth disturbance unrelated to climate, such as knotholes, were excluded from further analysis to avoid noise due to non-climatic effects (*n* = 29, corresponding to on average 2.4 excluded sampled trees per micro-site). Consequently, only trees with both TRW and MXD series available were used for the subsequent analysis (*n* = 319). All series were truncated after 2013 to exclude effects from the bark/coring process on MXD (cores were taken in 2014 and 2016) and to ensure that the same period was used for all sites and for both TRW and MXD. Cross-dating was verified and series were standardized using the dplR-package (Bunn, 2008) in the software environment R version 3.5.1 (R Core Team, 2018). We used a 30-year cubic smoothing spline with a 50% frequency cut-off to remove trends unrelated to climate, such as age and stand effects (Cook et al., 1990). Tree chronologies were prewhitened to remove autocorrelation and subsequently averaged into site chronologies for each micro-site using the biweight robust mean. Well-established descriptive statistics serving as quality criteria for the homogeneity of a tree population in dendrochronology, such as the expressed population signal (EPS), mean interseries correlation (rbar) and Gleichlaeufigkeit (glk), were calculated for all micro-sites and both proxies (Eckstein and Bauch, 1969; Wigley et al., 1984).

### Climate Data

Monthly resolved temperature means, precipitation sums and the SPEI (standardized precipitation evaporation index, Vicente-Serrano et al., 2010; integrated over 1, 3, and 6 months) were obtained for each site from the CRU TS 3.22 to 3.24 datasets at 0.5° resolution (Harris et al., 2014) for the respective nearest grid and the period 1901–2013. This CRU data was compared with the respective climate data from the nearest meteorological station obtained from the NOAA global database^2^. Due to strong correlations between modeled and station data for the overlapping period (0.97 to 0.99 for temperature and 0.85 to 0.97 for precipitation) and the overall shorter and sometimes discontinuous record of station data (station records start in 1936–1977, depending on the respective site) we decided to use the CRU dataset for climate-growth analysis in this study.

Averaged over the 1901–2013 CRU-data period, July and January were the warmest and coldest months, respectively, at all sites (except at Finnish forest sites where the mean temperature of January and February was identical), with the July temperatures being 0.5–1.3°C higher in the forest than at treelines and 1.2–2.1°C higher in Russia than in Finland. The smaller temperature range between the coldest and warmest months in Finland compared to Russia points to maritime influence in Finland. July precipitation sums are similar across sites (Table 2).

**Table 2:** Mean T_Jul, T_JA, and sum of P_Jul during the two climate regimes.

Region	Site	T_Jul wet/cold (°C)	T_Jul dry/warm (°C)	T_JA wet/cold (°C)	T_JA dry/warm (°C)	P_Jul wet/cold (mm)	P_Jul dry/warm (mm)
FIN	TD, TW	11.1	13.0	10.3	11.7	86	47
	FD, FW	12.5	14.5	11.7	13.1	83	46
RUS	TD, TW	13.0	15.6	12.1	13.9	67	47
	FD, FW	14.1	17.1	13.0	15.0	77	45
SIB	TD, TW	13.4	15.5	12.6	13.6	89	41
	FD, FW	13.6	16.0	12.8	14.2	87	44

*The same climate data was used for neighboring dry and wet micro-sites as they were located within one 0.5° CRU grid. Site abbreviations: T, treeline; F, forest; D, dry; W, wet*.

### Statistical Analyses

**FIGURE 2:**
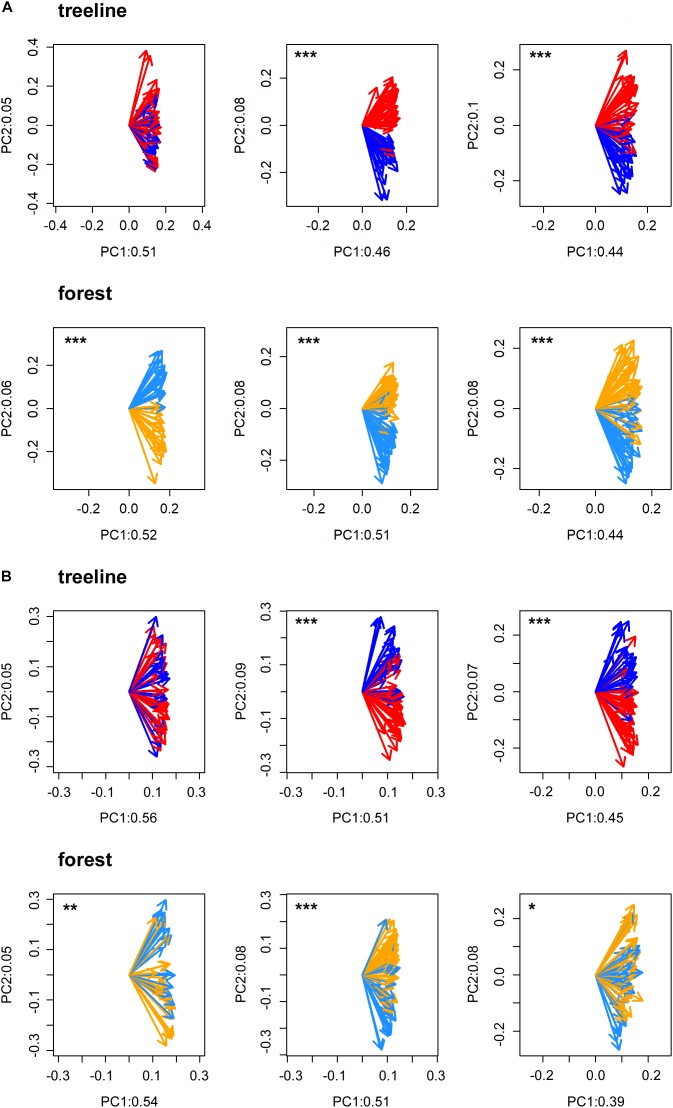
PCGA clearly separated the loadings of dry (treeline: red arrows and forest: orange arrows) and wet (treeline: dark blue arrows and forest: light blue arrows) micros-sites for tree-ring width **(A)** and maximum latewood density **(B)** in FIN (left), RUS (middle) and SIB (right). Labeling values of the principal components (PC) refer to the amount of total variance explained by the respective PC. Significant separations of loadings according to micro-sites are indicated with asterisks (^∗^*p* < 0.05, ^∗∗^*p* < 0.01, ^∗∗∗^*p* < 0.001).

All statistical analyses were performed in the R software environment version 3.5.1 (R Core Team, 2018). First, to statistically validate the selection of micro-sites and to test for possible heterogeneity within micro-sites, a Principal Component Gradient Analysis (PCGA, Buras et al., 2016) was performed with the standardized tree-ring series of each pair of dry and wet micro-sites. PCGA makes use of the loadings as derived from the first two principal components of an ordinary PCA to define a gradient of similarity among trees. To test whether the PCGA-loadings indicate micro-site specific growth signals, we applied a Wilcoxon rank-sum test. That is, we tested the similarity of locations (i.e., the non-parametric mean) of the loadings’ polar-coordinates between the respective micro-sites. A significant test indicates a true location shift (i.e., difference of non-parametric means) between the PCGA-loadings of the different micro-sites. Based on empirical evidence, PCGA is able to identify micro-sites expressing differing climate–growth relationships correctly (Rehschuh et al., 2017; Buras et al., 2018). Due to high accordance between micro-sites defined in the field and those identified by the PCGA for TRW (93% of the IDs were correctly assigned to their micro-site on average, Figure 2), we assumed that the differentiation between micro-sites was justified and that micro-sites were homogenous in themselves.

In order to identify the climate proxies and months with the strongest effect on radial tree growth at our micro-sites, we calculated Pearson correlation coefficients between micro-site chronologies and each climate proxy (i.e., temperature, precipitation and the SPEI) and month from June of the previous year until September of the year of growth for the period 1901–2013, using the R-package treeclim (Zang and Biondi, 2015). Micro-site chronologies were additionally correlated with seasonal summer temperature values expressed as the mean of June–July (JJ), July–August (JA) and June–August (JJA) temperatures. Stationary bootstrapping (Politis and Romano, 1994) was applied to all climate–growth correlations. For further analysis, we selected July temperature (hereafter T_Jul; for TRW) and the mean of JA temperature (hereafter T_JA; for MXD), both of the year of growth, at all sites as these were the climate proxies with the strongest signal at most of the sites.

To explore whether and to what extent differing climatic conditions prevailing before and during the growing season affect summer temperature–growth relationships, the period of 1901–2013 was divided into dry/warm and wet/cold years (hereafter called climate regimes; for the R code of the following analysis steps please refer to Supplementary Data Sheet 1). To this aim, the monthly water balance was calculated and averaged for April–July for each year of the period 1901–2013. Water balance was calculated by subtracting the potential evapotranspiration (PET) from precipitation. PET was calculated using the Thornthwaite equation (Thornthwaite, 1948) in the R-package SPEI (Beguería and Vicente-Serrano, 2017). Subsequently, the period of 1901–2013 was divided into years with a high and a low water balance, respectively, based on the aforementioned April-July mean. Due to significant positive Pearson correlations with precipitation (0.52 to 0.76; *p* < 0.001) and significant negative correlations with temperature (-0.49 to -0.58; *p* < 0.001) of the same April-July mean, years with a water balance above its 1901–2013 median were defined as wet/cold climate regime, while years with a water balance below its 1901–2013 median were defined as dry/warm climate regime accordingly. The classification of these two climate regimes was carried out for each site separately in order to consider the respective climatic span; a global classification of climate regimes valid for all sites was not viable due to insufficient overlap between sites. For subsequent analysis we selected the 25% most extreme years per climate regime and site (i.e., the 25% driest/warmest and 25% wettest/coldest years per site), resulting in 28 years per climate regime and site. This was done to restrict the analysis to the most extreme years but at the same time keep the sample size reasonably high. Years of both climate regimes were equally distributed across the period 1901–2013 at all sites. Across all sites, summer temperature was 1.9–3.0°C (T_Jul) and 1.0–2.0°C (T_JA) higher in dry/warm years compared to wet/cold years (Table 2). July precipitation was overall 20–48 mm lower in dry/warm years, corresponding to 46–70% of the precipitation falling in cold/wet years (Table 2).

Finally, Pearson correlation coefficients were calculated for the relationships between TRW and T_Jul, and between MXD and T_JA, by selecting only those years from the TRW and MXD chronologies and the respective climate data that were assigned to the dry/warm and the wet/cold climate regimes. In this way, correlations were performed separately for (i) years assigned to the dry/warm climate regime vs. years assigned to the wet/cold climate regime and (ii) for dry vs. wet micro-sites. Again, stationary bootstrapping (Politis and Romano, 1994) was applied. Additionally, Pearson correlations for the same combinations of micro-sites and climate regimes were performed for individual trees in order to investigate the variability of the summer temperature signal within micro-sites. In order to test if correlations differed significantly between micro-sites and between climate regimes, a pairwise Wilcoxon rank-sum test was carried out using correlation values of the individual tree-ring series. Tests were restricted to pairs where at least one correlation score was significant. The Holm correction for adjusted *p*-values was applied (Holm, 1979).

## Results

### Climate Signals

**FIGURE 3:**
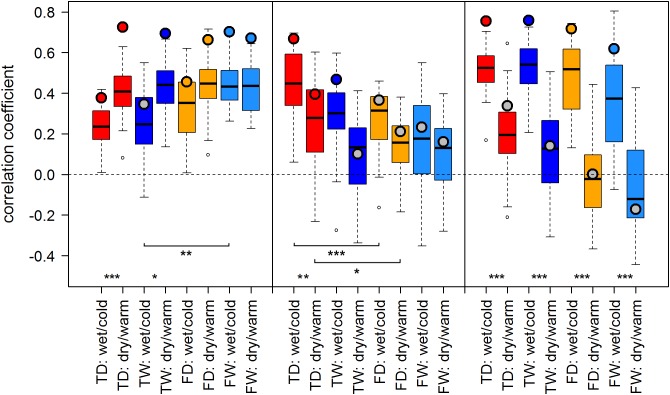
Temperature-growth correlations between MXD and T_JA are shown for each micro-site under dry/warm and wet/cold climate regimes for FIN (left), RUS (middle), and SIB sites (right). Boxplots represent the correlation values of individual trees of a micro-site. Colored circles represent the correlation value of the respective site chronology. Color code: red: treeline dry (TD); dark blue: treeline wet (TW); orange: forest dry (FD); light blue: forest wet (FW). Insignificant correlations of site chronologies are represented by a gray circle. Significant differences between dry/warm and wet/cold climate regimes, dry and wet micro-sites and between treeline and forest are marked with an asterisk (^∗^*p* < 0.05, ^∗∗^*p* < 0.01, ^∗∗∗^*p* < 0.001).

**FIGURE 4:**
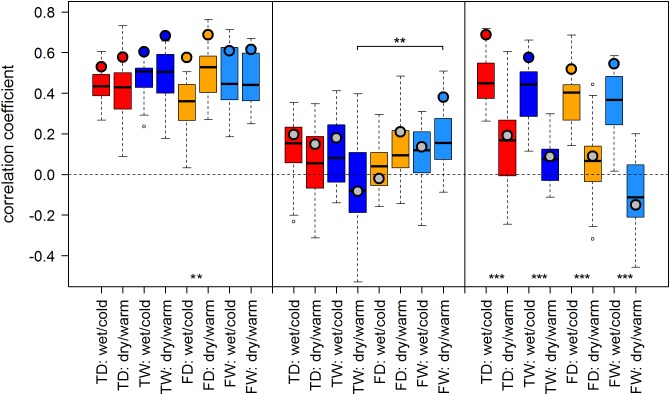
Temperature-growth correlations between TRW and T_Jul are shown for each micro-site under dry/warm and wet/cold climate regimes for FIN (left), RUS (middle), and SIB sites (right). For details, please refer to Figure 3.

Both proxies, TRW and MXD, showed generally similar results: Correlation scores were similar in sign and of comparable strength across most sites and climate regimes. We observed a tendency of somewhat stronger temperature–growth correlations for TRW in Finland and for MXD at RUS and SIB sites when compared to the respective other proxy. Overall, summer temperature signals were strongest in Finland and Western Siberia and weaker to non-significant in the north of European Russia (Figures 3, 4).

In the majority of cases, correlation values of site chronologies were stronger than the median of correlations of the individual trees at that site. The range covered by correlation values of individual trees slightly differed between micro-sites, climate regimes and the two proxies TRW and MXD without following a clear pattern, but with a tendency of higher variability for SIB MXD compared to TRW.

It is worth mentioning that correlations with precipitation over the whole 1901–2013 period, even though by far weaker than correlations with summer temperature, were significantly positive for May of the year of growth for TRW in Finland (up to *r* = 0.36, data not shown). Correlations with precipitation and the SPEI1, 3 and 6 over the whole 1901–2013 period were mostly significantly negative for the summer months (June to August of the year of growth) at FIN and SIB sites (but almost never at RUS sites) for MXD and sometimes TRW (data not shown).

### Effect of the Climate Regime

The effect of dry/warm and wet/cold climate regimes was strongest at all Siberian sites (Figures 3, 4): While correlations were strong and significant in wet/cold years, they were non-significant in dry/warm years. This climate signal loss between the two climate regimes in Western Siberia was significant for all micro-sites and results were consistent for both proxies. In Finland, correlation strength between dry/warm and wet/cold years varied marginally for TRW and stronger for MXD: It was stronger in dry/warm years and weaker in wet/cold years. However, variations between the two climate regimes were significant in only two micro-sites for MXD and one micro-site for TRW (Figures 3, 4). At the central site (RUS), only MXD at the dry treeline site showed significant climate signals differing between the two climate regimes with a pattern similar to SIB sites.

In terms of absolute growth, on average, TRW was larger and MXD was higher in dry/warm years than in wet/cold years at all sites.

### Effect of Micro-Sites and Treeline-Forest

Even though micro-site chronologies were clearly separated into dry and wet sites by the PCGA (Figure 2), significant differences regarding the summer temperature signal were not found between micro-sites. However, it is worth to mention that even though differences were insignificant, some correlation values were appreciably lower on wet compared to dry micro-sites (particularly for MXD at RUS treeline sites for both climate regimes). The comparison of treeline-forest revealed significant differences in four cases: Correlation strength was significantly higher at the dry treeline than at the dry forest site for MXD in RUS, in both climate regimes, exemplified by dry/warm and wet/cold years (Figure 3). Conversely, correlations were significantly higher at wet forest sites compared to wet treeline sites, for FIN MXD during wet/cold years and for RUS TRW during dry/warm years.

In terms of absolute growth, on average, TRW was larger on dry sites independent of the presence and direction of significant age differences (Tables 1, 3). Further, dry site trees were generally significantly thicker (except at Finnish treeline sites) and taller than wet site trees. Glk for both TRW and MXD was slightly higher on dry than on wet micro-sites at all but one of the Russian sites (Table 3).

**Table 3:** Tree metadata and descriptive statistics of TRW and MXD chronologies per micro-site.

Region	Site	dbh (cm)	Tree height (m)	Mean TRW (mm)	glk		EPS		rbar	
					TRW	MXD	TRW	MXD	TRW	MXD
FIN	TD	41.2	13.3^∗∗∗^	0.91	0.73	0.71	0.97	0.97	0.61	0.54
	TW	38.1	11.4^∗∗∗^	0.84	0.73	0.72	0.96	0.96	0.54	0.53
	FD	49.6^∗∗∗^	21.1^∗∗∗^	0.70	0.69	0.67	0.96	0.95	0.58	0.49
	FW	35.3^∗∗∗^	13.6^∗∗∗^	0.58	0.70	0.69	0.96	0.97	0.62	0.64
RUS	TD	37.7^∗∗∗^	17.3^∗∗∗^	1.22	0.70	0.73	0.97	0.97	0.50	0.49
	TW	26.0^∗∗∗^	11.6^∗∗∗^	0.68	0.65	0.67	0.97	0.92	0.67	0.42
	FD	34.8^∗∗∗^	15.4^∗∗∗^	0.92	0.72	0.68	0.98	0.97	0.50	0.47
	FW	26.5^∗∗∗^	13.7^∗∗∗^	0.82	0.68	0.64	0.98	0.94	0.64	0.36
SIB	TD	34.8^∗^	12.1^∗∗∗^	1.08	0.66	0.68	0.99	0.96	0.75	0.46
	TW	31.2^∗^	9.6^∗∗∗^	0.70	0.66	0.67	0.97	0.96	0.57	0.48
	FD	38.5^∗∗∗^	13.8^∗∗∗^	0.68	0.67	0.64	0.95	0.94	0.41	0.36
	FW	24.6^∗∗∗^	10.8^∗∗∗^	0.65	0.65	0.63	0.96	0.95	0.45	0.40

*Glk, EPS, and rbar were calculated for the period that is relevant for climate-growth analysis (1901–2013). Dbh and tree height were tested for significant difference between each pair of dry and wet micro-sites using a Wilcoxon rank-sum test or a Student’s *t*-test (depending on whether data was normally distributed). Significant differences are marked with an asterisk (^∗^*p* < 0.05, ^∗∗^*p* < 0.01, ^∗∗∗^*p* < 0.001). Site abbreviations: T, treeline; F, forest; D, dry; W, wet*.

Summarized, in terms of temperature-growth correlations five out of twelve or 42% (TRW) to seven out of twelve or 58% (MXD) of the micro-sites were significantly affected by shifts of the growing season climate regime, while only one (TRW) to three (MXD) micro-site pairs were significantly affected by the difference between treeline and forest, and no temperature-growth correlation was significantly affected by micro-site differences.

## Discussion

In this study, we clearly demonstrate that the strength of the summer temperature signal in Scots pine is primarily affected by inter-annually differing climate regimes at our sites, while ecological micro-site conditions play a minor role. More particularly, growing season climate variability led to significantly different temperature–growth correlations at about half of our sites (mostly in Russia), while differing micro-sites significantly affected absolute tree growth, but not the climatic signal. We discuss possible reasons for this differentiated reaction to growing season climate in space and time and evaluate the role of micro-site conditions.

### Inter-Annual Climate Variability Drives Spatially Differing Tree-Growth Responses to Climate

Coupled to large-scale circulation systems, regional climate is undergoing natural inter-annual variations in temperature and precipitation within a certain range. Trees as living but immobile organisms are able to adapt to these variations to a certain degree by reducing or enhancing radial growth under unfavorable or favorable climatic conditions, respectively (Fritts, 1976).

The present study shows explicitly that as a consequence of this growth adaptation to climate, the temperature signal preserved in annual growth rings varies accordingly across years and has a spatially differentiated pattern. At most sites in Russia (RUS and SIB), wet/cold years seem to be growth limiting, leading to narrow annual rings, a low MXD and a strong summer temperature signal. Correspondingly, dry/warm years promoted radial growth at Russian sites, leading to wider rings, a high MXD and a weak or absent summer temperature signal. This temperature-dependent growth pattern fits well with the growth limitation hypothesis for near-treeline locations (Körner, 1998, 2012; Holtmeier, 2009), even though it is surprising that this inter-annual variation of climate regimes seems sufficient to trigger significantly different growth reactions. It is unlikely that this reduction in summer temperature signal in dry/warm years was caused by drought stress, unlike similar studies (e.g., Seo et al., 2011; Düthorn et al., 2013), as ring growth was enhanced (larger TRW) during dry/warm years and correlations with summer precipitation were either significantly negative or absent when correlated over the whole 1901–2013 period. Since neither temperature nor precipitation seem to be growth limiting here in dry/warm years, other factors might become growth limiting here, such as, e.g., competition for light and nutrients (Fritts, 1976).

Correspondingly, we would have expected the same pattern, i.e., a stronger summer temperature signal in wet/cold than in dry/warm years also in Finland. However, we found that the climatic signal between the two climate regimes differed significantly only at few sites in Finland, being stronger during dry/warm years in contrast to Russia. As summer temperatures and correlation strength were similar between wet/cold years in Russia and dry/warm years in Finland (Table 2) – this was possible because the two climate regimes have been defined relative per site – we conclude that Scots pine growth is limited by similar thermal conditions in Russia and in Finland. Regarding the differences in correlation strength between the two climate regimes in Finland particularly found for MXD at treeline, wet, cold, and cloudy conditions during the growing season might have led to a reduction of the summer temperature signal in wet/cold years as reported in previous treeline studies, e.g., due to a late onset of cambial activity caused by late snowfall and late snowmelt (Kirdyanov et al., 2003), a reduced photosynthetic productivity caused by high precipitation rates/high cloud cover (Düthorn et al., 2016), or changed hydrological conditions in the soil (Linderholm et al., 2014).

In contrast to MXD, ring-width formation is particularly affected by climatic conditions of the early growing season (Rossi et al., 2011, 2012), generally occurring between mid-May and mid-June in the area of our Finnish forest sites (Rossi et al., 2008; Seo et al., 2008, 2011). Significant positive correlations of TRW with May precipitation over the 1901–2013 period suggest a high importance of water availability during the early growing season at our Finnish sites, which is corroborated by findings of Seo et al. (2011) in the same area. This positive effect of wet conditions in spring might counteract the negative effect of wet/cold conditions during summer months, possibly explaining the slightly stronger summer temperature signal in wet/cold years in TRW compared to MXD in Finland.

The effect of differing climatic conditions was strongest at the easternmost site (SIB) and weakest in Finland for both tree-ring proxies, as initially hypothesized. This is possibly related to absolutely higher summer temperatures occurring at the more continental Russian sites (Table 2), which are located further east and south than the Finnish sites, following the northern distribution limit of Scots pine (Figure 1 and Table 1). Growth limiting conditions are possibly more easily and more strongly exceeded in Russia, while in Finland both climate regimes seem to be closer to growth limiting climatic conditions (Table 2). Higher summer temperatures in Russia might further explain the low to insignificant correlation values at all central (RUS) sites. Even though not being the southernmost location in this study, RUS forest sites exhibited the highest summer temperatures among all forest sites under both climate regimes (Table 2), which possibly lowers the effect of temperature–growth limitation. The reason for the missing temperature signal in TRW at RUS treeline sites remains unclear so far and needs further investigation.

### Growth Adaptation to Ecological Site Conditions Hardly Interferes With the Summer Temperature Signal

Unlike previous assessments of micro-site effects on Scots pine radial growth (e.g., Moir et al., 2011; Düthorn et al., 2013; Linderholm et al., 2014; Matskovsky and Helama, 2014), ecological site conditions only had minor effects on the summer temperature signal of Scots pine in this study, suggesting that possible ecological effects were largely removed during the standardization process. Different standardization methods might lead to different results when comparing micro-site effects though (Düthorn et al., 2013, 2015), with the choice of the most appropriate standardization method for the respective dataset being one of the main quality criteria for climate reconstructions (Esper et al., 2016).

However, tree height and dbh were significantly reduced on wet sites. This growth adaptation was likely caused by unfavorable and stressful growing conditions, such as anaerobic soils and low nutrient availability, and is in line with other studies on Scots pine from peatlands (e.g., Moir et al., 2011; Smiljanić et al., 2014) or wet sites (Düthorn et al., 2013, 2015). Just as in similar studies (e.g., Smiljanić et al., 2014; Blanchet et al., 2017), we experienced difficulties in cross-dating ring parameters from trees growing at wet sites due to frequently occurring narrow and missing rings and found a slightly lower agreement of tree-ring series (i.e., glk) within wet micro-sites in Russia (e.g., Linderholm et al., 2014; Edvardsson et al., 2015; Nöjd et al., 2017) in both TRW and MXD. Even though the PCGA clearly identified differing ecological conditions between pairs of dry and wet micro-sites, comparability between micro-sites across regions and with other micro-site studies cannot be assured.

### Implications and Outlook

We showed that differing climate regimes significantly impacted the temperature signal of Scots pine at about half of our sites, for both TRW and MXD, overriding micro-site effects on the temperature signal. With the significant weakening of the summer temperature signal in Scots pine under certain climatic conditions (particularly in Western Siberia under dry/warm climate regimes) our findings contribute to the divergence phenomenon and loss of temperature sensitivity discussion (D’Arrigo et al., 2008). Although the temperature signal overall was slightly stronger in MXD (T_JA) than in TRW (T_Jul), both tree-ring proxies were similarly affected by the loss of temperature sensitivity in our study. Since reconstructions of summer temperature based on TRW and MXD rely on the assumption that radial tree growth is limited by temperature and that this growth limitation is stable over time, the suitability of the present dataset for dendroclimatological applications such as temperature reconstructions remains to be tested, for both TRW and MXD (but see Tuovinen et al., 2009). Even though we hardly found significant effects of micro-site differences on the summer temperature signal at our sites, we noticed a tendency of weaker correlations at most Russian wet sites.

Concluding, we recommend a thorough examination of possible instabilities stemming from past climate fluctuations and ecological site differences despite an apparently stable signal before attempting temperature reconstructions. An evaluation of micro-site effects might be particularly relevant for the calculation of global vegetation and carbon cycle models, where absolute growth is important. Depending on the respective interplay of temperature and precipitation, the projected future increase of both climate proxies at higher latitudes (IPCC, 2014) might have regionally differing consequences for the future climatic signal of near-treeline trees, but will likely be dominated by temperature increase, thus probably resulting in a reduction of the summer temperature signal in Scots pine in those areas of the Eurasian boreal forest that are most affected by global warming.

## Data Availability Statement

Datasets are available on request: the raw data supporting the conclusion of this manuscript will be made available by the authors, without undue reservation, to any qualified researcher.

## Author Contributions

JL, MW, and J-WS designed the study with input from MG, RJ, and VK. JL, RC-G, MG, RJ, VK, and MW collected the data and assisted JL with measurements. JL analyzed the data with input from AB and MW. AB performed the PCGA. JL wrote the first draft of the manuscript with contributions of MW. All authors contributed to manuscript revision, read, and approved the submitted manuscript.

## Conflict of Interest Statement

The authors declare that the research was conducted in the absence of any commercial or financial relationships that could be construed as a potential conflict of interest.

## Footnotes

1 www.walesch.ch

2 https://www.ncdc.noaa.gov/
